# Focal Non-aneurysmal Subarachnoid Hemorrhage After Carotid Artery Stenting: A Case Report

**DOI:** 10.7759/cureus.62104

**Published:** 2024-06-10

**Authors:** Shimpei Tsuboki, Takamasa Mizuno

**Affiliations:** 1 Neurosurgery, Ariake Medical Center, Arao, JPN; 2 Neuroendovascular Surgery, Kameda Medical Center, Kamogawa, JPN

**Keywords:** carotid artery stenosis, endovascular treatment (evt), cerebral hyperperfusion syndrome, subarachnoid hemmorhage, carotid artery stenting (cas)

## Abstract

Minor non-aneurysmal subarachnoid hemorrhage (SAH) following carotid artery stenting (CAS) is exceedingly rare and less described, with its underlying mechanism elusive. Here, we present the case of a 75-year-old female who underwent CAS for progressive asymptomatic severe stenosis of the internal carotid artery. Her post-procedural course remained uneventful, with no intracranial hemorrhage detected on the following day's magnetic resonance imaging (MRI). However, a routine MRI on the seventh post-procedural day identified a small amount of SAH in the central sulcus on the operative side. In the absence of symptoms, the patient was discharged home after a computed tomography (CT) scan revealed no signs of hemorrhagic enlargement the following day. In this report, we document the rare occurrence of localized SAH post-CAS. There are limited reports of minor SAH following CAS, with the underlying mechanisms remaining unclear. In this report, the localization of SAH aligns with the most critical ischemic sites, indicating that the mechanism of focal SAH after CAS is associated with blood-brain barrier (BBB) disruption due to a rapid increase in blood flow to small vessels with impaired vascular autoregulation. Focal convexity SAH is an easily overlooked finding, and the medical team performing carotid artery revascularization procedures should be aware of the potential for such SAH postoperatively and exercise caution during postoperative imaging interpretation.

## Introduction

Intracranial hemorrhage after carotid artery revascularization is regarded as a manifestation of cerebral hyperperfusion syndrome. Notably, subarachnoid hemorrhage (SAH) is particularly uncommon among such cases, especially non-aneurysmal SAH [1], the mechanism of which remains elusive. Herein, we present a case of non-aneurysmal SAH following carotid artery stenting (CAS) and elucidate its etiology through a comprehensive review of the existing literature.

## Case presentation

**Figure 1:**
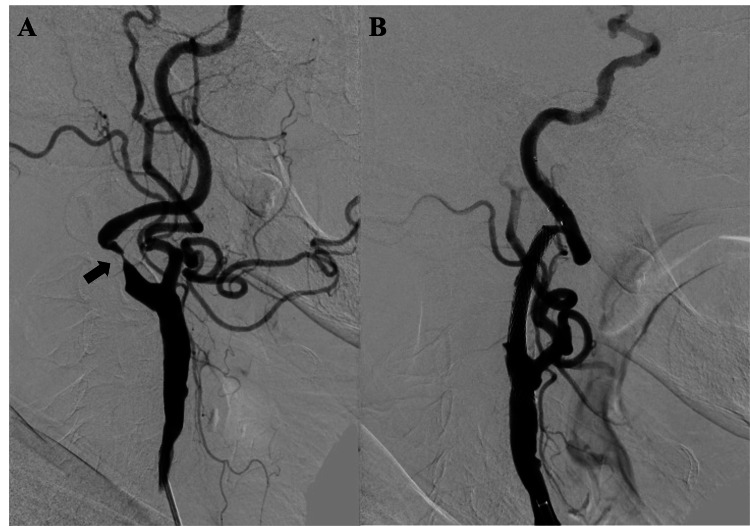
Digital subtraction angiography of the neck before and after carotid artery stenting Lateral images of common carotid angiography before and after carotid artery stenting of the neck (A, B) showed that severe internal carotid artery stenosis (arrow) was dilated after stenting.

A 75-year-old female presented at our department due to progressive right internal carotid artery (ICA) stenosis evidenced by increasing peak systolic velocity (PSV) on serial ultrasonography examinations. She remained neurologically intact without any deficits. Her medical history included hypertension, diabetes mellitus, hypercholesterolemia, and chronic thoracic aortic dissection. Diagnostic digital subtraction angiography (DSA) confirmed 80% stenosis of the right ICA according to the North American Symptomatic Carotid Endarterectomy Trial (NASCET) mode (Figure 1A), accompanied by sluggish intracranial blood flow. The Matas test revealed no collateral circulation through the anterior communicating artery. No abnormalities were noted in the intracranial vasculature. Aspirin and clopidogrel were initiated two weeks prior to the procedure. Right CAS via a brachial artery approach was performed under local anesthesia. She was given heparin IV to achieve a periprocedural-activated clotting time of over 250 seconds. Thereafter, a 6-Fr FUBUKI Dilator kit (ASAHI Intecc, Japan) was advanced into the right common carotid artery and the stenosis was crossed with a Filter Wire EZ (Boston Scientific, Marlborough, MA, USA). The stenosis was predilated with a Starling 3.0mm × 40mm catheter balloon (Boston Scientific). A carotid wall stent 10 × 24mm (Boston Scientific) was then placed in the stenosis and post-dilated with a Starling 5.0mm × 20mm catheter balloon (Boston Scientific) (Figure 1B).

**Figure 2:**
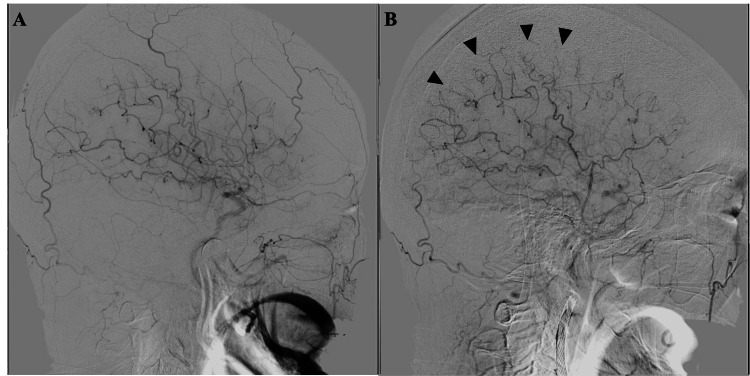
Digital subtraction angiography of the head before and after carotid artery stenting Lateral images of common carotid artery angiography pre- and post-dilation of the head (A, B) revealed enlargement of the perfusion area in the right middle cerebral artery region and depicts peripherally dilated small arteries after stenting (arrowhead).

There were no complications (i.e., slowing or stoppage of blood flow, embolism of distal vessels, or dissection) as assessed by periprocedural angiography during the procedure. Following post-dilation, DSA demonstrated an improvement in cerebral blood flow retardation, with enhanced delineation of the middle cerebral artery (MCA) extending more peripherally (Figures 2A, 2B).

**Figure 3:**
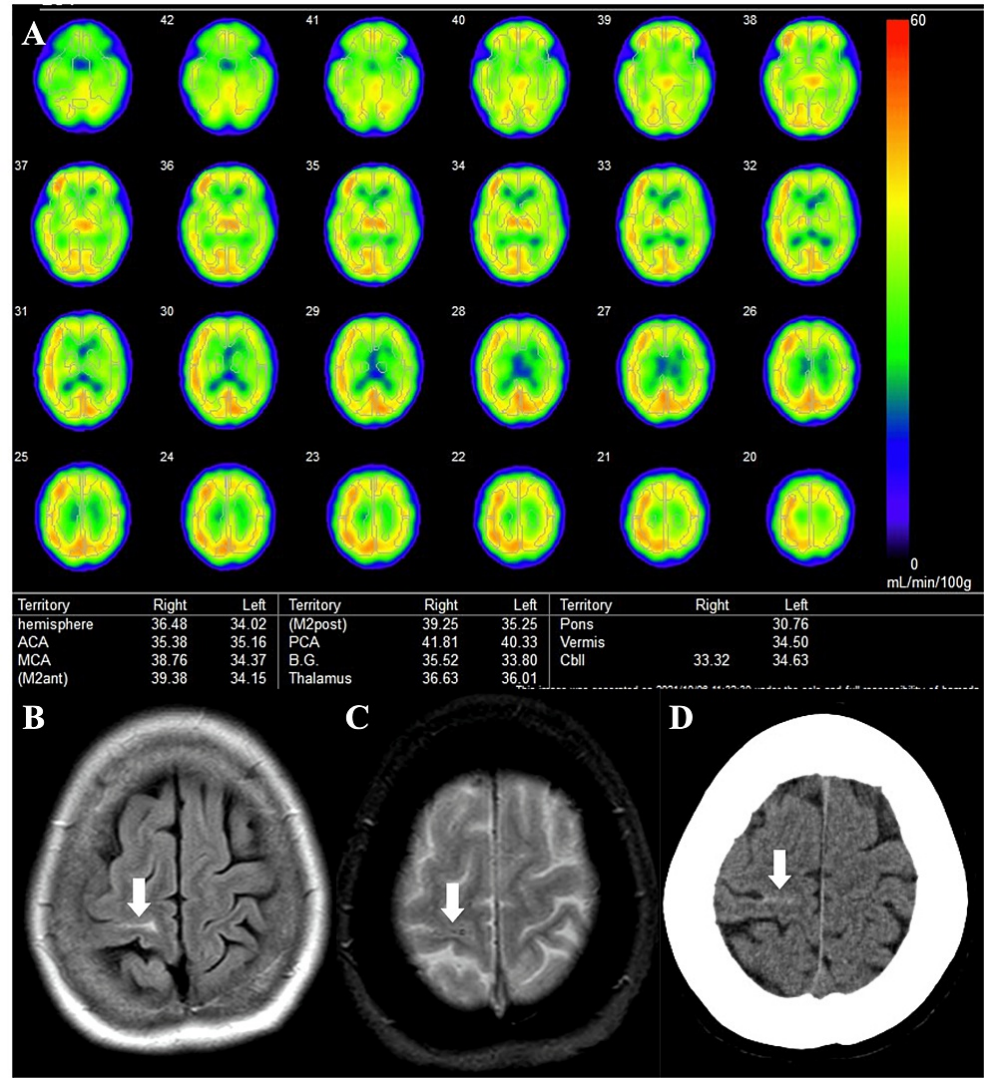
Radiological imaging after carotid artery stenting Quantitative cerebral blood flow measurements using N-isopropyl-(iodine 123) p-iodoamphetamine and single photon emission computed tomography (SPECT) on two days after carotid artery stenting (A) showed no obvious increase in cerebral perfusion on the operative side. Fluid attenuated inversion recovery (FLAIR) imaging (B) and T2*-weighted image (C) on magnetic resonance imaging (MRI) performed seven days after carotid artery stenting and computed tomography (CT) imaging eight days after stenting (D) showed small amount of subarachnoid hemorrhage in her right central sulcus (arrow).

Subsequent magnetic resonance imaging (MRI) conducted a day post-operation revealed minimal spotty diffusion-weighted imaging (DWI) positive lesions in her right basal ganglion and insula cortex. And the MRI also showed no evidence of hemorrhage. Quantitative cerebral blood flow assessments utilizing N-isopropyl-(iodine 123) p-iodoamphetamine and single photon emission computed tomography (SPECT) performed two days post-procedure showed no significant cerebral hyperperfusion, but a 10% increase in perfusion on the left side compared to the right (Figure 3A). Strict blood pressure control was continued for one week and the patient's postoperative course remained uneventful. Routine MRI performed on seventh postoperative day unveiled SAH within the right convexity sulcus (Figures 3B, 3C), although the arterial spin labeling perfusion imaging showed no evidence of increased cerebral perfusion. No neurological deficits or headaches were observed in the patients. As the hemorrhage was minor, the dual antiplatelet therapy was continued. A computed tomography (CT) scan performed eight days post-operation (Figure 3D) revealed no enlargement of the SAH. Consequently, the patient was discharged home on that day devoid of any symptoms.

## Discussion

SAH after carotid artery revascularization is exceedingly rare; its occurrence following carotid endarterectomy (CEA) remains uncertain, estimated to comprise approximately 10% of intracranial hemorrhages following CAS [1]. The majority of SAH cases post-carotid artery revascularization is linked to intracranial aneurysms, with the frequency and etiology of non-aneurysmal SAH yet to be determined.

Historically, several instances of non-aneurysmal SAH have been documented post-CEA and CAS [2,3]. These occurrences predominantly manifest in cases of severe stenosis, with diffuse SAH, such as in the basal cisterns and Sylvian fissures, being more prevalent. The prognosis, particularly in CAS cases, tends to be unfavorable. The perioperative administration of multiple antiplatelet agents and anticoagulants may exacerbate post-bleeding outcomes in CAS procedures, as has been reported with increased hemorrhagic complications due to clopidogrel hyper-response [4]. Concerning the timing of bleeding, occurrences post-CEA typically present later, suggesting a potential association with hyperperfusion syndrome. However, there exists only one report each of cortical SAH post-CEA [2] and CAS [5], and it remains ambiguous whether this condition aligns with massive SAH.

Two mechanisms of hemorrhage following carotid artery revascularization have been proposed: the first involves microembolization induced during the operation, resulting in hemorrhagic infarction; the second entails disruption of the blood-brain barrier (BBB) due to the influx of large volumes of blood into micro-vessels with compromised autoregulation owing to chronic hypoperfusion [1,6]. In this case, the stenotic lesion was severe, and a comparison of pre- and post-stenting images revealed enlargement of the perfusion area on the lesion side, accompanied by a watershed shift before and after stenting. This area was presumed to have the most hypoperfused and impaired vascular autoregulation, consistent with the localization of the SAH. This observation lends support to the second mechanism of hemorrhage. Another condition known to induce cortical SAH similar to the present case is reversible cerebral vasoconstriction syndrome (RCVS), wherein disruption of the BBB is purported to underlie the occurrence of SAH [7], thereby further substantiating the second mechanism of hemorrhage. Regarding treatment and prognosis, the patient in this report received strict blood pressure management, leading to favorable outcomes. Previous reports have documented cases of transient neurological deficits even in the presence of cortical SAH [4], underscoring the necessity for blood pressure control as in the case of cerebral hyperperfusion syndrome when hemorrhage is detected.

This report has certain limitations. It must be acknowledged that although no significant cerebral hyperperfusion was observed during the examination, the possibility that the patient experienced hyperperfusion at the onset of SAH cannot be excluded, nor can the potential over-efficacy of antiplatelet drugs be disregarded. The pathogenesis of SAH remains speculative.

## Conclusions

In this study, we have presented a case of focal convexity SAH subsequent to CAS. While the etiology and pathological implications of this condition remain elusive, it manifests in the same region where the watershed shift was observed, implying hemorrhage resulting from heightened cerebral blood flow exposure to small vessels with diminished vascular autoregulation. The medical team undertaking carotid artery revascularization procedures should remain cognizant of the potential occurrence of such SAH postoperatively and exercise caution during postoperative imaging interpretation.
